# Cereal Domestication and Evolution of Branching: Evidence for Soft Selection in the *Tb1* Orthologue of Pearl Millet (*Pennisetum glaucum* [L.] R. Br.)

**DOI:** 10.1371/journal.pone.0022404

**Published:** 2011-07-22

**Authors:** Marie-Stanislas Remigereau, Ghayas Lakis, Samah Rekima, Magalie Leveugle, Michaël C. Fontaine, Thierry Langin, Aboubakry Sarr, Thierry Robert

**Affiliations:** 1 Laboratoire Ecologie, Systématique et Evolution, Université Paris-Sud XI, Orsay, France; 2 Institut de Biotechnologie des Plantes, Université Paris-Sud XI, Orsay, France; 3 Université Pierre et Marie Curie, Paris, France; University of Umeå, Sweden

## Abstract

**Background:**

During the Neolithic revolution, early farmers altered plant development to domesticate crops. Similar traits were often selected independently in different wild species; yet the genetic basis of this parallel phenotypic evolution remains elusive. Plant architecture ranks among these target traits composing the domestication syndrome. We focused on the reduction of branching which occurred in several cereals, an adaptation known to rely on the major gene *Teosinte-branched1* (*Tb1*) in maize. We investigate the role of the *Tb1* orthologue (*Pgtb1*) in the domestication of pearl millet (*Pennisetum glaucum*), an African outcrossing cereal.

**Methodology/Principal Findings:**

Gene cloning, expression profiling, QTL mapping and molecular evolution analysis were combined in a comparative approach between pearl millet and maize. Our results in pearl millet support a role for *PgTb1* in domestication despite important differences in the genetic basis of branching adaptation in that species compared to maize (e.g. weaker effects of Pg*Tb1*). Genetic maps suggest this pattern to be consistent in other cereals with reduced branching (*e.g.* sorghum, foxtail millet). Moreover, although the adaptive sites underlying domestication were not formerly identified, signatures of selection pointed to putative regulatory regions upstream of both *Tb1* orthologues in maize and pearl millet. However, the signature of human selection in the pearl millet *Tb1* is much weaker in pearl millet than in maize.

**Conclusions/Significance:**

Our results suggest that some level of parallel evolution involved at least regions directly upstream of *Tb1* for the domestication of pearl millet and maize. This was unanticipated given the multigenic basis of domestication traits and the divergence of wild progenitor species for over 30 million years prior to human selection. We also hypothesized that regular introgression of domestic pearl millet phenotypes by genes from the wild gene pool could explain why the selective sweep in pearl millet is softer than in maize.

## Introduction

**Figure 1:**
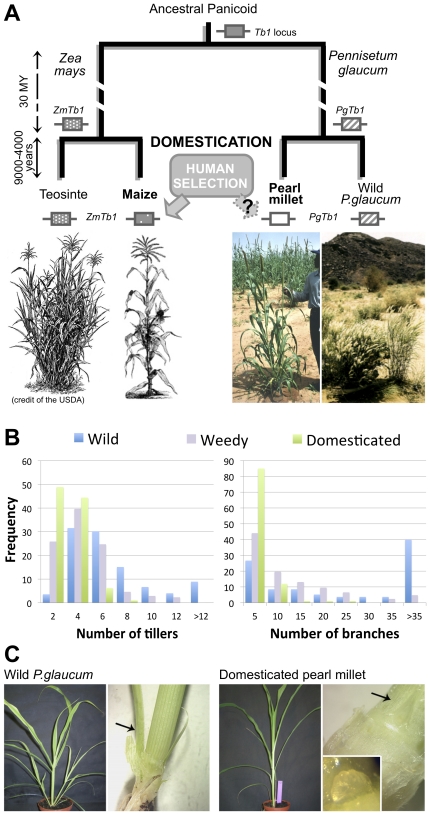
Hypothesis of parallel genetic evolution at the *Tb1* locus for the adaptation of vegetative branching during maize and pearl millet domestication. A. The phylogenetic tree shows that *Zea mays* and *Pennisetum glaucum* are two wild grasses from the Panicoid sub-family that separated 30 million years ago (dotted lines, scale not respected), wild *Z.mays* (teosinte) growing in America and wild *P.glaucum* in Africa. About 9,000–4,000 years ago, they were independently domesticated into maize and pearl millet, respectively. Pictures below the tree illustrate the parallel morphological evolution of both wild progenitors during their domestication, in particular the reduction of tillering and branching. *Z.mays* and *P.glaucum* inherited from their most recent common ancestor the orthologous copies *ZmTb1* and *PgTb1*of the developmental gene *Tb1* (represented by a hatched box). It was previously shown that *ZmTb1* has been targeted by human selection for the reduction of maize branching during domestication. We ask whether *PgTb1* was subjected to parallel evolutionary processes for the similar adaptation of branching during the domestication of pearl millet. B. Distribution of the number of tillers and branches in domesticated pearl millet, wild *P.glaucum* and weedy plants grown in the same location in south Niger. The cultivated field and the wild population were in parapatric situation. Plants present in the field were classified as domestic or as weedy according to farmer's classification. The ability of weedy pearl millets to shed their seeds spontaneously at the maturity stage is one of the main factors used by farmers to recognize them [13]. Histograms show that domestication was associated with a reduction of vegetative branching. These data were obtained on more than 200 plants for the wild and the domestic pearl millets repectively, and more than 150 plants for weedy phenotypes. C. Tillering in young *P.glaucum* seedlings. At 4 weeks after germination, tillers are visible in wild *P.glaucum* (left) but not in the Souna domesticated landrace (right). Close-ups after dissection reveal that axillary meristems have developed into an emergent tiller in the wild plant (arrow) but remain dormant as buds with 1 or 2 leaves (arrow) or undeveloped meristems (box) in the cultivated landrace.

Plant domestication provides many examples of repeated phenotypic evolution [1], [2] and a powerful system to explore its genetic basis [3], [4]. Cereals in particular share many common adaptations to cultivation which define the domestication syndrome [1], [2]. They were domesticated from different wild grass species in distinct domestication centers 10,000 to 4,000 years ago [5]. Human selection focused on the seed and shaped the generally small-sized, naturally dispersed and coated wild seed into the typical cereal grain, large, naked, devoid of dormancy and dispersal ability [5], [6]. In maize, sorghum and millets (cereals of the *Panicoideae* subfamily), the characteristic bushy architecture of wild progenitor species was also altered and branch number strongly reduced [7]. In maize in particular, vegetative branching was almost completely suppressed (Figure 1A). The genetic dissection of these developmental adaptations in crosses between domesticated crops and their respective wild relatives has revealed that, despite the multigenic inheritance of domestication traits, some of the underlying quantitative trait loci (termed domestication QTL) coincide at conserved syntenic locations in the different cereal genomes. This has prompted the hypothesis that man could have unconsciously and independently selected the same repertoire of genes for the domestication of multiple species. This would constitute a large scale process of parallel genetic evolution [1], whereby repeated phenotypic evolution proceeded by the recurrent, independent emergence and selection of new adaptive mutations at homologous genes (reviewed in [8], [9], [10], [11]).

This initial view has been refined as the genetic control of developmental traits targeted by domestication is gradually revealed in model systems like rice and maize. Prior to domestication, wild progenitor grass species have diverged over 65 million years during which they have strongly diversified morphologically through the evolution of gene networks. It is now clear that many of these networks control the same developmental traits as those later targeted by domestication [7]. For some traits, genes have conserved their role in different grass species and sometimes even across monocots and dicots. In contrast, for other traits, developmental gene networks have evolved specifically in such a way that key determinants differ in related species (e;g. the *ramosa* gene in Panicoideae, which cannot be found in rice despite extensive efforts to clone it) [7]. Positional cloning of domestication genes is still tedious, slowing the advances to identify these determinants and compare them across species. Therefore, the hypothesis of parallel genetic evolution during domestication is not trivial and needs to be tested directly by a candidate-gene approach for a given domestication gene.

Like in maize, vegetative branching has been considerably reduced during pearl millet domestication (*Pennisetum glaucum*) [12] (Figure 1). Even though branch number still segregates in domesticated pearl millet populations, cultivated varieties produce much less branches than wild *P.glaucum* (Figure 1A–B). In some areas, segregation of wild features in the domesticated gene pool may be due to the occurrence of weedy plants, which display intermediate branching phenotypes [13] (Figure 1B). Previously, we reported a domestication QTL for reduced vegetative branching in this species which covered a region predicted to harbor the *Teosinte-branched1* (*Tb1*) gene according to comparative mapping [12], [14]. *Tb1* is a plant-specific transcription factor [15] and a major domestication gene in maize [16]. While the barley *Tb1* orthologue has recently been shown to contribute to spike architecture differences between two-rowed and six-rowed varieties [17], *Tb1* has mainly been associated to the development of vegetative branches. Its specific targets and mode of action are yet unknown but transgenic and mutant studies of *Tb1* homologs in rice, sorghum and *A.thaliana* showed that it contributes to repress the activity of vegetative axillary meristems where it is expressed, and their expansion into branches [18], [19], [20], [21]. Vegetative branching is a very complex and highly multigenic trait requiring the coordination of meristem growth by multiple pathways, including local meristematic and long-distance hormonal signals from roots and shoots, as well as environmental cues (reviewed in [22], [23]). Surprisingly, *Tb1* was singled out as the only major gene involved in the adaptation of vegetative branching during the domestication of maize, accounting for 35% of the trait variance [16], [24], [25], even though stem number is controlled by at least 8 other loci in the wild progenitor teosinte [26]. Further studies revealed that human selection targeted adaptive sites located upstream of the gene, possibly in regulatory sequences related to a hypothetic dosage effect of *Tb1* on development or to the strong pleiotropy of the gene over inflorescence structure [27], [28].

*Tb1* is an obvious *a priori* candidate gene for the adaptation of vegetative branching in other domesticated species due to its conserved function in the control of this trait in dicots [19] and monocots (grasses) [18], [20]. However, it has never been formally proven to be involved in the evolution of branching during domestication other than in maize. In fact, patterns of evolution in the coding sequence of the gene suggest that changes in the TB1 protein did not contribute to the morphological diversification of grasses [29]. This does not preclude the eventuality of positive selection on other *Tb1* regions, such as its regulatory sequences. In the single study published to date examining *Tb1* roles in the evolution of tillering during domestication other than in maize [30], a cDNA clone of the maize *Tb1* gene was shown to coincide with a domestication QTL in some foxtail millet crosses (*Setaria italica*). This QTL was minor and its effects considerably smaller than those of *Tb1* in maize (9% *vs* 35% on average). Therefore, *Tb1* effects seem to vary greatly between species, making it difficult to predict if the gene may be a “key” locus recurrently recruited for the evolution of branching during domestication. The ontogeny of axillary stems from different types of vegetative meristems (see first section of results and ref.[7]), as well as the pleiotropy of *Tb1* on inflorescence architecture [16], [17], [24], [25] are further *a priori* arguments against the possibility of parallel genetic evolution at this locus.

In this study, we asked if the *Tb1* locus played a role in the evolution of tillering in pearl millet, using a candidate-gene approach to investigate the parallel evolution observed between maize and pearl millet during their domestication (Figure 1A). To test this hypothesis in the absence of routine transgenic technology in non-model species, we first checked whether polymorphism in the gene segregates with branching variation in pearl millet genetic crosses. We also extended this survey to rice, sorghum and foxtail millet. Secondly, we verified that *Tb1*'s expression pattern is conserved in pearl millet. Thirdly, we tested whether sequence polymorphism at the *Tb1* locus in domesticated and wild populations is consistent with a recent event of human selection. For these purposes, we cloned *PgTb1*, the orthologue of *Tb1* in pearl millet and used a combination of QTL mapping, expression and molecular evolution analyses.

## Results

### Comparative QTL mapping for vegetative branching in cereals

Grasses produce two types of axillary stems from their main primary shoot. Tillers are issued at a basal position, from nodes that are put in place early during seedling development, and they often develop their own adventitious roots independent from the main stem. Branches grow from nodes located higher up on the stem, after this latter starts elongating (after flowering induction) [7]. Both types of branching have been reduced in domesticated sorghum [7], foxtail millet [30], maize [25] and pearl millet [12] (Figure 1A). Absence of branching at a node can arise from various developmental defects related to different genetic networks [23]. The vegetative axillary meristem can either fail to initiate at the axil of the leaf, as observed in some foxtail millet varieties [7], or it can be arrested in its organogenic activity, like it is the case in maize [31], sorghum [20] and foxtail millet [7]. To investigate whether it is so in pearl millet as well, we dissected domesticated and wild plants at different stages of development. As illustrated in Figure 1C, tillers and branches fail to develop in domesticated plants due to the arrested activity of their vegetative axillary meristems which remain dormant either as meristems or as small buds with one or two leaf primordia. Therefore, branching adaptation during domestication has comparable developmental origins in maize, sorghum, foxtail and pearl millet, and could be caused by orthologues of the same genes involved in axillary meristem activity.

**Figure 2:**
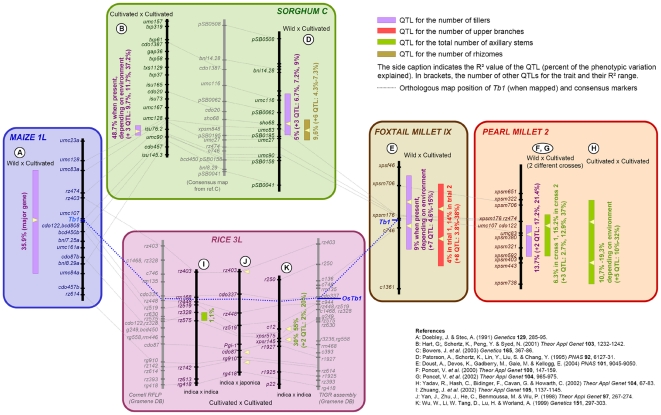
Comparative mapping of domestication QTLs for vegetative branching in cereals. The orthologous map segments syntenic to the maize *Tb1* region are aligned following consensus markers (linked by dotted grey lines). QTLs associated to branching are indicated by their confidence intervals (colored boxes). The respective percent phenotypic variance they explain (R^2^) is reported alongside the number and effects of other QTLs in the same cross. These QTLs tend to be consistently conserved at similar positions around the mapped or predicted location of *Tb1* orthologues in sorghum, foxtail millet and pearl millet, and in some rice crosses involving parents with contrasted vegetative branching architecture.

By assembling a comprehensive comparative genetic map of QTLs for axillary branching in these four species (Figure 2), we observed that QTLs for branching reduction are consistently detected in the predicted region for *Tb1* in sorghum and pearl millet, in addition to the previously described cases of association with the gene in maize [16] and foxtail millet [30]. These QTLs reflect adaptation of branching during both domestication (in “wild progenitor x cultivated landrace” crosses) and secondary crop diversification (in crosses between varieties). Interestingly, the *Tb1* region of perennial species of sorghum also harbors QTLs for the production of rhizomes (Figure 2), which are structurally equivalent to underground tillers [7]. On the other hand, the *Tb1* region is not associated to domestication QTLs in wheat (not shown) or rice (Figure 2), although transgenic experiments have shown that *Tb1* orthologues of these species have conserved a role in tiller development [18]. This is consistent with the fact that the vegetative architecture of pooids (wheat) and ehrhartoid (rice) cereals was not altered by domestication. Instead, they produce a profuse number of tillers (and no upper branches), like their wild progenitors [7]. However, QTLs for tiller number map close to *OsTb1* in crosses involving rice varieties that have been specifically selected for a low-tillering phenotype during secondary crop diversification (Figure 2).

This comparative map also revealed that the genetic basis of branching adaptation during the domestication of sorghum and millets is in sharp contrast with maize. It involves multiple genes in addition to *Tb1*, some of which have much stronger effects on the trait than *Tb1* (Figure 2). As opposed to observations in maize, *Tb1* effects in those species are usually moderate to low and sometimes depend on environmental conditions (e.g. in foxtail millet [32]). Altogether, these results suggest a consistent pattern of parallel evolution of vegetative branching in cereals based in part on the repeated selection of *Tb1*, despite strong differences from a species to another in the respective contribution of *Tb1* orthologues to the genetic basis of domestication.

### Characterization of *PgTb1* in *Pennisetum glaucum*

**Figure 3:**
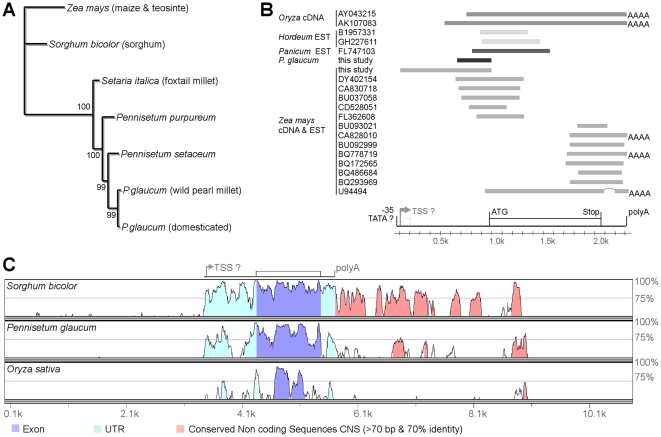
Structure and sequence conservation of grass *Tb1* orthologues. A. Phylogenetic tree built from the alignment of *Tb1* orthologous sequences (from start to stop codon) using maximum likelihood. Bootstrap values at the nodes were estimated from 500 replicates. B. The position of orthologous *Tb1* EST and cDNA from grass species identified by a nBLAST search of the Genbank database are reported relative to the maize *ZmTb1* gene. In the schematic representation of the structure of *ZmTb1*, white boxes stand for exons, lines for UTR and introns. The putative transcription start site (TSS) is indicated by an arrow and the box in interrupted lines is a putative short exon reported by [33] but not supported by any of the EST and cDNA data. C. Analysis of pairwise sequence conservation between *ZmTb1* (BAC clone AF464738) and the orthologous regions of sorghum (AF466204), rice (AC091775) and peal millet using the VISTA software [51]. Evolutionary conserved regions (ECR or CNS) were defined by a sliding window analysis with a threshold size of 70 bp and a minimum 70% nucleotide identity.

We first isolated the homologous *Tb1* coding sequence by polymerase chain reaction (PCR) in pearl millet (*P.glaucum*) and other wild *Pennisetum* species. In an approach similar to Lukens & Doebley's in [29], we used primers from conserved regions of the gene (Table S1) to isolate *Tb1*-like sequences. The product we cloned shared strong nucleotide and amino acid identity with *Z.mays Tb1* (87% and 83% respectively), especially in the specific TCP and R transcription factor domains. Southern blotting (not shown) indicated that this gene was present as a single copy in *P.glaucum,* in agreement with previous studies in *Andropogoneae* [29]. This, along with the high identity levels and the phylogenetic tree built from the aligned *Tb1* sequences (Figure 3A), demonstrated that the gene we isolated in pearl millet is orthologous to maize *Tb1* (the *P.glaucum* gene is hereafter referred to as *PgTb1* and the *Z.mays* orthologue as *ZmTb1*). Reverse transcription PCR (RT-PCR) on seedling RNA also confirmed that *PgTb1* is a functional gene expressed in wild and domesticated *P.glaucum*.

The structure of *Tb1* has been somewhat unclear due to a putative cryptic intron reported in *ZmTb1* [33]. We examined the expressed-sequence tag (EST) and cDNA sequences homologous to *ZmTb1* and available in grasses, and found all of them to be perfectly colinear with the corresponding DNA sequences (Figure 3B). RT-PCR was also performed with several sets of conserved primers which failed to detect any splice variant in *ZmTb1* or *PgTb1* (e.g. on Figure S1). This confirmed that *Tb1* is a single-exon gene in both maize and pearl millet. These RT-PCR results located the transcription start site roughly 900bp upstream of the start codon (Figure 3B). This structure is probably conserved in other *Tb1* orthologues according to the EST and cDNA data available in other cereals.

To gain a view of sequence evolution in the *Tb1* genomic region, we isolated a bacterial artificial chromosome (BAC) containing *PgTb1* and compared it to orthologous BACs from maize, rice and sorghum. While conservation between *Tb1* orthologues was high in their transcribed region (>73% nucleotide identity on average), similarity dropped abruptly upstream of the transcription start site (alignment was impossible in those regions). In contrast, many highly conserved non-coding sequences were found up to 9kb downstream of the gene, some of them shared by all four orthologues (Figure 3C).

**Figure 4:**
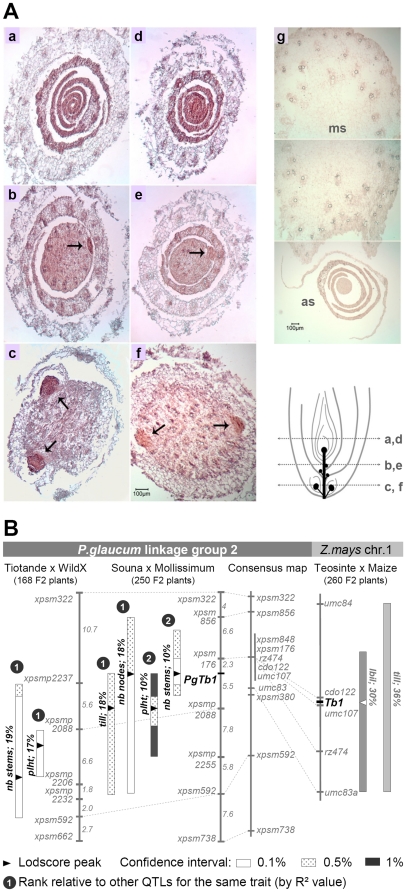
Conservation of *PgTb1* function during plant development. A. *In-situ* hybridization of the *PgTb1* mRNA in serial transverse sections of a 10 day-old seedling of (a–c) the Souna landrace, (d–f) a 10 day-old wild seedling and (g) at the upper node of a mature wild plant (ms: main stem; as: axillary branch). Positions of the sections are given on the bottom-right diagram. B. Association of *PgTb1* with domestication QTLs for tillering and branching in two domesticated x wild crosses. Souna and Tiotandé are landraces from Niger and Senegal, respectively. We mapped *PgTb1* in the reference cross 81b x icmp451 to position the gene on the consensus pearl millet genetic map. The corresponding *ZmTb1* locus and associated maize domestication QTLs are taken from ref. [24]. The percentage of variation explained by a QTL (R^2^) is reported beside its confidence interval (box). Abbreviations: *till*, tiller number; *nb stems*, total number of stems (tillers & branches); *nb nodes*, node number on the main stem; *plht*, plant height; *lbil*, average branch internode length on the main stem.

We also examined the timing and location of *PgTb1* expression during pearl millet development. In greenhouse conditions, wild and domesticated plants began to differ at 25 to 30 days after germination (8–10 visible leaves). At that stage, the first tiller became visible and most axillary meristems at other nodes had developed buds with one or two leaf primordia in wild seedlings, while these meristems remained dormant and did not produce buds in domesticated plants (Figure 1C). Time series of RNA *in-situ* hybridizations detected *PgTb1* transcripts in the axillary meristems as early as 10 days after germination (Figure 4A). Results were identical in 30 day-old plants. After floral induction, *PgTb1* was also expressed in axillary meristems and buds at upper nodes along the main stem, albeit at lower levels than at basal nodes, especially in wild plants (Figure 4A). The gene was not expressed in other organs than vegetative axillary meristems. These expression patterns were similar to those reported for *Tb1* orthologues in maize, rice and *A. thaliana* [18], [19], [31] and supported the hypothesis that *PgTb1* has conserved its function in the control of vegetative axillary meristem growth in *P.glaucum*.

### Association of *PgTb1* with domestication QTLs

To test comparative mapping predictions, we refined QTL detection after mapping *PgTb1* and additional flanking markers in the same wild x cultivated crosses as we had previously analyzed (Figure 4B). Among other QTL segregating around the gene, *PgTb1* was the lodscore peak marker for a QTL controlling the total number of stems (log likelihood ratio of 34.1). The *PgTb1* allele contributed by the domesticated parent was associated with fewer tillers and branches. In both crosses, the effects of the gene on the trait were modest (10% or 18% phenotypic variance explained) and they were complemented by at least four other QTL of equivalent effects. Even though direct transformation to rule out potential effects of other linked genes is yet impossible in *P.glaucum*, these results strongly suggested that *PgTb1* underlies a domestication QTL for the reduction of axillary vegetative growth.

### Patterns of positive selection in *PgTb1*

If *PgTb1* is a domestication gene, it should display signatures of a recent selective sweep [3], [4], [34]. We analyzed sequence polymorphism in 6.7kb across *PgTb1*, in a wide collection of 52 accessions representing the diversity of wild and cultivated *P.glaucum* (Figure S2 and Table S2). Polymorphism was compared between *PgTb1* and three single-copy sequence tagged-site loci (STS) located on different linkage groups and away from domestication QTLs. They provided a control for neutral evolution in contrast with the human selection that occurred during domestication. A search of Genbank using BLASTn indicated that STS 713 is likely coding and shares high similarity with multiple plant protein kinases from the RLG family in maize, sorghum and rice (e-values 3e-69 to 4e-04), while STS 476 shared similarity with an expressed mRNA of unknown function in Sorghum (e-value 3e-165) and STS 738 was non-coding (one single hit in rice with e-value 6e-07).

**Figure 5:**
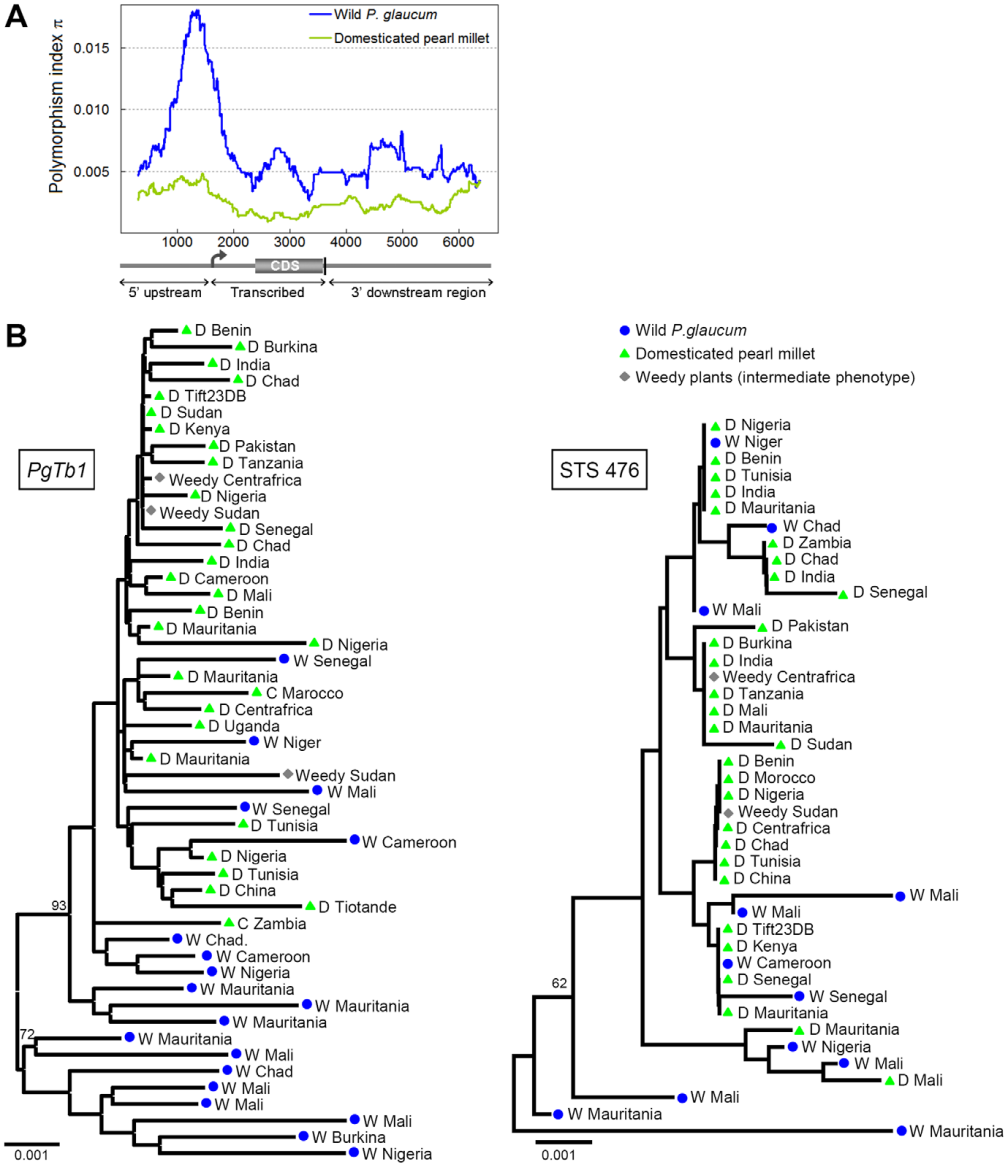
Molecular polymorphism at the *PgTb1* locus. A. Sliding-window plot of the polymorphism index π in the *PgTb1* region. Values were calculated separately in wild (blue) and domesticated (green) samples in a 600 bp window. B. Genetic tree of *PgTb1* alleles (right) and for one of the STS loci (left), constructed using the neighbor-joining method and the Kimura-2P distance (gaps excluded). Significant bootstrap support is indicated at the node and was calculated for 1,000 random permutations.

The polymorphism indices π and θ measure nucleotide diversity and are reduced by genetic sampling during population bottleneck as well as by positive selection, two processes characteristic of domestication. Population bottleneck affects the whole genome while selection effects are restricted to the targeted loci and regions in linkage disequilibrium with them. The ratio of π_cultivated_/π_wild_ showed that cultivated pearl millet is 46% less polymorphic than wild *P.glaucum* across all loci on average (Table 1) which is consistent with a recent bottleneck. *PgTb1* sequences also lost two times more diversity than the STS loci (60% *vs* 32% on average), possibly reflecting an additional event of selective sweep in *PgTb1*. This loss of genetic diversity in domesticated plants was uneven throughout the *PgTb1* region, as illustrated by a sliding window plot of π values (Figure 5A). The strong difference between domesticated and wild polymorphism levels was particularly visible upstream of the of the transcription start site and within the transcribed region, where the drop in diversity reached 70% (Table 1). These results suggested an action of selection on those regions.

**Table 1:** Genetic diversity and tests of selection in *PgTb1* and STS control loci.

		Sample size a	Length	S b	Singletons	Specific sites c	θ d	π d	Dom/Wild π ratio (%)	Tajima's D	Tajima's D *p*-value (%) ‡	Fu & Li's F*	Fu & Li's F* *p*-value (%) ‡
***PgTb1*** ** upstream**	Wild	19	1573	86	58	70	0.016	0.010		−1.439	5.80	−2.232	4.20
	Dom.	29	1587	58	45	41	0.009	0.004	34	−2.333	0.03<p<0.18	−3.736	0.19<p<0.65
***PgTb1*** ** mRNA**	Wild	19	1860	64	43	56	0.010	0.005		−1.916	1.50	−2.399	7.30
				*nc*∶33			*nc*∶0.013	*nc*∶0.006					
				*syn*∶12			*syn*∶0.013	*syn*∶0.011					
				*nsyn*∶19			*nsyn*∶0.006	*nsyn*∶0.003					
	Dom.	29	1865	34	29	26	0.005	0.002	32	−2.341	0.04<p<0.57	−4.008	0<p<0.61
				*nc*∶16			*nc*∶0.005	*nc*∶0.002					
				*syn*∶7			*syn*∶0.007	*syn*∶0.002					
				*nsyn*∶11			*nsyn*∶0.003	*nsyn*∶0.001					
***PgTb1*** ** downstream**	Wild	19	2666	95	73	82	0.010	0.005		−1.986	0.70	−2.913	1.10
	Dom.	29	2692	96	88	79	0.009	0.003	57	−2.548	0<p<0.04	−4.707	0.<p<0.02
**STS 713** **	Wild	18	1160	31	15	26	0.008	0.006		−0.654	27.12	−0.994	16.94
	Dom.	18	1161	21	14	16	0.005	0.003	50	−1.480	3.8<p<7.10	−2.014	4.00<p<7.90
**STS 738** **	Wild	13	1970	42	24	24	0.007	0.006		−0.791	21.73	−1.153	15.44
	Dom.	17	1976	37	16	21	0.006	0.005	94	−0.090	24.<p<52.9	−0.544	13.10<p<30.3
**STS 476** **	Wild	12	727	13	8	10	0.006	0.004		−1.112	13.18	−1.209	15.37
	Dom.	29	768	7	2	2	0.002	0.002	51	−0.106	25.7<p<71.5	−0.124	16.9<p<75.9

a The same collection was sequenced for the STS loci and *PgTb1.* When some STS loci could not be amplified in a given individual (for technical reasons), they were sequenced in an individual from a geographically close population (details in Table S2), in order to keep sample size and composition consistent with *PgTb1* and between STS. Individuals Tb70, Tb83 and Tb84 that are issued from seeds collected in wild populations (see Table S2) displayed a weedy-like phenotype at maturity. For this reason, we preferred to discard their sequences from the wild sample for the genetic diversity analysis. Conclusions from neutrality tests were unchanged when these 3 plants were included (data not shown).

b S: number of segregating sites.

c Specific sites: sites polymorphic in one population (e.g. wild) but monomorphic in the other (e.g. domesticated).

d θ: Watterson estimator of sequence diversity, π: average number of differences per site between two sequences. For both estimators, the diversity in the *PgTb1* mRNA region is broken down into non-coding (nc), synonymous (syn) and non-synonymous (nsyn) sites.

‡ For the domesticated sample, a p-value of D and F* was calculated for each one of the 54 simulated scenarios of demographic history. This table gives the range of these p-values excepting scenario 39 which was discarded on the basis of its extremely low likelihood (see methods and Figure S3C): significant *p*-values consistent across all demographic scenarios provide strong support for selection. Refer to Table S3 for a comprehensive list of these *p*-values per scenario and per region (details of the scenarios and simulation methods in Figure S3).

**STS 713 is on linkage group 6, STS 476 on linkage group 3 and STS 738 on linkage group 2 at 27 cM from *PgTb1* (referred to as marker Xpsm738 on the genetic map of Figure 4B).

To test this hypothesis, we implemented several tests of neutrality on the basis of DNA sequence polymorphism data. We first performed an HKA test [35] by using STS 476 as a control locus. Only STS 476 was used since it was the only adequate neutral, coding locus (single BLAST hit (sequence XM 002450113.1) *vs* multiple possible homologues for STS 713). The alignment between the Sorghum *Tb1* sequence (AF466204) and *PgTb1* limited the HKA test to a short ∼400 bp stretch of the 5′ upstream region, the ORF and ∼350 bp of the 3′ downstream region. Both on this whole region and on the coding sequence only, the HKA tests resulted not significant either in the domesticated or the wild sample (data not shown).

We also implemented two other tests of neutrality, namely Tajima's D [36] and Fu & Li's F* [37]. Population size of wild *P.glaucum* was supposed large and constant, with no effect on D and F* statistics. However, the population expansion following the strong bottleneck experienced by the domesticated population could be a cause for an excess of rare alleles (e.g. singletons) as equally as a recent selective sweep. This would translate into negative D and F* values due to demography rather than positive selection. Therefore, we generated the expected distributions of D and F* under conditions of population bottleneck and subsequent expansions consistent with the history of domestication and the archaeological record [38], [39] (see Methods for details). Several combinations of model parameters were tested to explore a wide range of possible demographic scenarios. The significance of the observed values of D and F* was tested against this modified null hypothesis of neutrality and demography (Figure S3).

The results of Tajima's D test and Fu & Li's F* neutrality tests are presented in Table 1 (see also Table S3 for more details). As expected, the control STS loci were compatible with expectations both of neutrality (with respect to natural selection in the wild sample) and absence of selection associated to domestication (Table 1). Only one locus (STS 713) was significant in the domesticated sample but only in some scenarios and only at the 5% level (p > 3.8% without correction for multiple testing). It could therefore not be excluded that STS 713 bears the weak signature of a past selective sweep in the domesticated sample (and maybe in both populations), possibly in relation to its coding nature as a RLG-like kinase.

In contrast with these STS loci, neutrality tests supported a highly significant selective sweep in domesticated pearl millet in the region upstream of *PgTb1*. D and F* values were highly significant regardless of the demographic scenario considered, in strong contrast with the corresponding wild *P.glaucum* sequences which were consistent with neutrality (Table 1). Neutrality was also rejected in the transcribed and downstream regions in the domesticated sample but it was harder to conclude as to a selective effect of domestication, since both regions also seem to be subjected to natural selection in wild P.*glaucum* (Table 1). In the absence of demographic information, it was impossible to explore whether this excess of rare alleles was due to a selective sweep or to a recent expansion of the wild population (the latter would also be in agreement with negative D and F* values observed for all loci including the STS in the wild sample).

Altogether, neutrality tests supported a signature of selective sweep related to domestication in *PgTb1,* markedly stronger upstream of the gene and in agreement with the sliding window analysis of sequence polymorphism as well as the domesticated-to-wild ratios of θ and π These results were consistent with the hypothesis that human selection acted on *PgTb1* and that the gene was involved in pearl millet domestication. They also pointed to a stronger selective sweep in the intergenic sequences upstream of the gene that mirrored previous results reported in maize *ZmTb1* [27]. However, the intensity of the selective sweep in *PgTb1* was noticeably less than in *ZmTb1*: while most of the wild genetic diversity is lost in maize [27], a third still segregates in pearl millet (Table 1). Possible explanations for this important discrepancy are discussed below. It could explain the failure of HKA tests to detect deviation from neutrality in *PgTb1*. Another reason could be the poor alignment scores with Sorghum, as well as the use of STS 476 for which low levels of polymorphism reduce the power of the HKA test.

The absence of fixation in domesticated pearl millet made it impossible to identify putative causal sites. However, in both pearl millet and maize *Tb1* orthologues, the footprint of human selection was centered on upstream regions theoretically involved in the regulation of the gene. Furthermore, our data suggested a possible footprint of natural selection in wild *P.glaucum*, which is not unexpected given that *PgTb1* indirectly controls inflorescence number, an element of plant fitness. This also suggested that the gene may have played a broader role in the evolution of grasses beyond solely cereal domestication, as formerly speculated [29].

## Discussion

A few years ago, Paterson *et al.* [1] proposed that the analogous morphological transformations induced by domestication to turn wild grasses into crops shared a common genetic basis. Our study addressed this hypothesis directly with molecular data for a candidate domestication gene. We argue that the same *Tb1* gene has been targeted by human selection in pearl millet and maize in relation to the similar adaptations of their vegetative branching pattern. Three observations are consistent with this hypothesis: (1) sequence and expression pattern conservation between *Tb1* orthologues (Figures 3 and 4A) indicate that the gene function was conserved since the divergence of *Zea* and *Pennisetum* and could therefore be a common target of human selection for similar adaptations in the two species; (2) *Tb1* orthologues segregate with vegetative branching variation in crosses between wild and domesticated plants (Figure 2 and 4B); (3) sequence polymorphism in the *Tb1* orthologue is consistent with evidence of human selection (Table 1 and Figure 5A).

Further investigations would help consolidate this conclusion. First, definitive proof of the implication of *PgTb1* in the reduction of tillering would require transformation of a domesticated plant with a wild *PgTb1* allele (and reciprocally). Second, tests of neutrality based on sequence polymorphism are limited by controls for demographic events in the history of the sample. We explored a wide range of bottleneck/population expansion scenarios, from the most extreme to the most compatible with archaeological evidence (Figure S3) but additional sequence data at neutral loci would help infer the precise demographic history of pearl millet.

Also, while we did not identify another gene or open-reading frame in the vicinity of *PgTb1* within the BAC clone that we sequenced (data not shown), the selective sweep pattern could result from selection on another distant region in linkage disequilibrium with the gene. Previous studies in maize were confronted with the same problem, for lack of fixation in domesticated populations [27]. It is strongly suspected that a combination of multiple adaptive sites is involved in maize, some of which have been fine-mapped to regions located between 58kb and 69 kb upstream of the selective sweep originally detected flanking *ZmTb1* [28]. Despite the very strong divergence we observed between *Z.mays* and *P.glaucum* in these intergenic regions, it is possible that short regulatory elements have been conserved in both species.

### Why is the signature of selection weaker in pearl millet than in maize?

The amplitude of the selective sweep associated to domestication (Figure 5A) is arguably lower in *PgTb1* than it is in *ZmTb1* [27]. Several theoretical studies have shown that such soft sweeps are in fact likely to be common and can stem from different alternative explanations [34], [40], [41], [42].

First, these two *Tb1* orthologues strongly differ in their respective contribution to the genetic basis of vegetative branching adaptation (oligogenic in maize but multigenic in pearl millet). In contrast with *ZmTb1*, the moderate-to-low and non pleiotropic effects of *PgTb1* may have prevented mutations from being counter-selected in wild progenitor populations prior to domestication. Therefore, adaptive sites may have been harbored in different initial *PgTb1* haplotypes responsible for the higher genetic diversity in domesticated landraces. Similar cases of cryptic variation at intermediate frequencies has been documented for several domestication traits in teosinte [43]. Such selection on standing genetic variation reportedly affects the intensity of selective sweeps [34], [40] and therefore the power to detect signatures of selection in domestication genes [34].

It is also unknown whether multiple wild progenitor populations may have contributed to pearl millet domestication [44]. Statistical support of *PgTb1* phylogenies did not point clearly to a unique origin of the domesticated sample (Figure 5B). In the case of a reintroduction and recombination of polymorphism from various wild populations during the domestication process, the interference between linked adaptive sites originally proceeding from different wild populations could have shaped a soft sweep [42].

Current agricultural practices in traditional areas of pearl millet cultivation could also prevent the fixation of *PgTb1* alleles. In previous studies, we reported that farmers traditionally proceed with selection in the granary, *i.e.* based on seed and panicle traits and taking no account of the vegetative branching or general architecture of the plants from which seeds were harvested [13], [45]. This would be of little consequence if the domesticated phenotype was fixed, but on the contrary, a significant phenotypic diversity often segregates in the fields (Figure 1B). Cultivated plants can range from typically “domesticated” to weedy types displaying shorter panicles, smaller seeds and significant vegetative branching [13], [45]. In the Sahel, where sympatry with wild *P.glaucum* still prevails, these intermediate phenotypes result from indeterminate generations of hybridization between domesticated, weedy and sometimes wild plants. They account for an important proportion of millet plants found in the fields [45] and despite their lower agronomical quality, they are harvested when other cultivated plants fail to withstand the aridity and unpredictable rainfall. This process whereby less-adapted individuals are selected if better competitors are rare is a typical case of “soft selection” [46] and it likely contributes recombinant *PgTb1* alleles to the domesticated gene pool. The impact of this long-term process extends far beyond regions of sympatry with wild *P.glaucum* because seeds are traded on a large geographic scale [47]. Domestication of pearl millet can therefore be considered as a still on-going process and strong selective sweeps cannot be achieved under these conditions. This could also explain why population genetics tests (D anf F*) were more efficient than HKA in detecting selection in our study. These tests are indeed known to be efficient for detecting ongoing selection acting on segregating variants [48].

This soft sweep in *PgTb1* is in agreement with the emerging re-evaluation of plant domestication as a process that may have taken place much slower than previously envisioned, at least for some phenotypes. For instance, archeological records of spikelets and rachis fossils demonstrated that the evolution of non-shattering forms in rice, barley and einkorn wheat was very slow [6]. It suggests that selection pressures for this trait were surprisingly weak during domestication, at least for these three cereals.

### Remaining issues and broader implications

First, a better understanding of the causal polymorphisms, of the regulation of *Tb1* expression and of the gene's function during plant development are required to elucidate the molecular mechanism of the adaptation of branching during domestication. Analyses of natural polymorphism extended to larger *Tb1* regions and accurate comparative measures of *Tb1* expression, in complement to already undertaken reverse genetics studies in the model species *A.thaliana* [19], [21] will likely contribute to fill this gap.

Second, did other domestication events? Studies in foxtail millet [30], comparative QTL maps (Figure 2) and preliminary expression data in sorghum (Figure S4) open the intriguing perspective that this hypothesis could extend to other cereals for which domestication also reduced branching. Moreover, the *Tb1* orthologue in barley has been shown to be involved in the differences of spike architecture between two-rowed and six-rowed varieties, probably through its already demonstrated role in kernel development in this species [17]. However, this hypothesis needs to be investigated further by systematic cloning, evaluation of *Tb1* effects and molecular evolution analyses in those other species.

What common properties of *Tb1* orthologues would make them preferential targets of human selection during distinct domestication events? Rapid phenotypic evolution such as that sought by domestication may require genes with significant effects while avoiding deleterious antagonistic pleiotropy [10], [11]. It has been proposed that transcription factors acting at lower-order levels of regulatory networks could therefore be predominantly involved in natural variation [49], [50]. Furthermore, case studies have illustrated that parallel morphological evolution seems to bias for selection on the control sequences upstream of transcription factors, not because of the strength of these mutations but rather because these genes are located at key positions in regulatory networks and act as integrators between upstream patterning genes and downstream structural effectors [10], [11]. *Tb1* could be just such a gene in plants, a hypothesis that will likely be tested as details of its mode of action and targets become available in model systems.

### Conclusions

The independent emergence of similar traits in distinct lineages is a common phenomenon observed at all taxonomic levels and this has long raised the fascinating question as to whether these repeated phenotypic changes evolve from similar or from different, unique genetic mechanisms. Plant domestication has led to strikingly similar morphological adaptations. In many cereals, it involved the modification of architecture by selecting for plants developing fewer branches. We examined the genetic basis of this adaptation by comparing pearl millet and maize, domesticated in Sub-Saharan Africa and Mexico respectively. Our study supports that domestication in pearl millet involved the same *Teosinte-branched1* gene as previously documented in maize. Genetic maps suggest this could have also been the case in other cereals with reduced branching, like sorghum. However, *Tb1* has modest effects in pearl millet in comparison with maize, and the changes in the branching habit of domestic plants have required other loci. Signatures of selection pointed to putative regulatory regions upstream of both *Tb1* orthologues in maize and pearl millet, suggesting that some level of parallel genetic evolution could explain the similar reduction of branching in these two crops. These results are unanticipated given the complex control of branch development, the multigenic inheritance of this domestication trait in pearl millet and the millions of years of morphological diversification of wild grass progenitor species prior to their domestication.

However, polymorphism patterns in *Tb1* orthologues also pointed to important differences between pearl millet and maize. For example, evidence of selection was found in the coding and 3′ downstream regions in both domesticated and wild pearl millet. In addition, we have shown that the selective sweep is much weaker in pearl millet than in maize. We suggest that this soft sweep is due to the ongoing and common introgression of the domesticated pool by wild and weedy pearl millet, a process we had previously documented in the Sahelian region [44], [45].

## Materials and Methods

All primers used for this study are reported in Table S1.

### Cloning of *PgTb1* and conservation with *Tb1* orthologues

Primer design in conserved regions of the gene was guided by homologous grass *Tb1* sequences retrieved from Genbank by a BLASTn search. In particular, L2 primer is a 25bp element downstream of *Tb1* found identical in 14 cDNA and EST sequences (AY043215, AK107083, BQ293969, BQ778719, BU093021, CA828010, AF543434-41). The sequence of the *PgTb1* product obtained was deposited in Genbank (AY631857). For Southern blotting, genomic DNA was digested with *BamHI* or *XhoI* prior to electrophoresis, blotted and probed with the U1/L2 *PgTb1* PCR product labeled with dCTP-alpha^32^P. We used the same probe to screen the Tift23DB BAC library from the John Innes Centre, Norwich, UK. A BAC clone was sub-cloned and sequenced by the John Innes Genome Centre. We assembled these sequences using the Staden package (http://staden.sourceforge.net). Evolutionary comparisons between BAC clones were conducted with the VISTA program [51].

### RT-PCR assays

RT-PCR was performed with SuperscriptII RT (Invitrogen) using 1 µg of total RNA extracted from maize and pearl millet seedlings, and the resulting products were sequenced. We also cloned the pearl millet *EF1-alpha* gene (*alpha* subunit of *elongation factor1*) to use as a control, since it is constitutively expressed and contains an intron. Consensus *EF1-alpha* primers were derived from available grass orthologous sequences.

### *In-situ* hybridization

Tillering strongly varies with stock and environment, therefore tissues were sampled from accessions previously selfed over 6-7 generations to reduce the effects of genetic background, and grown in replicate under controlled-cabinet conditions (30°C, 12 hours of day, spaced by 25 cm). Fixation, embedding, labeling and hybridization were performed following Hubbard *et al.* [31]. Replicate hybridizations of a given accession yielded identical results and patterns were similar in different domesticated landraces (Ligui from Chad and an early flowering landrace from Senegal). Control hybridization with *EF1-alpha* was strong and homogeneous across sections.

### Mapping and QTL detection

We mapped *PgTb1* in the 81b x icmp451 reference cross using a CAPS marker (cleaved amplified polymorphic sequence) typed by *TaqI* digestion and polyacrylamide electrophoresis of the U1/L1 PCR product. In the Souna x Mollissimum cross, *PgTb1* was mapped using a single-strand conformation polymorphism (SSCP) in the U1/L1 product, and SSR markers [52] were also added to improve map coverage. In the Tiotande x wildX cross, the lack of an easily typed marker impaired *PgTb1* mapping but four SSR markers were added to enable comparative mapping. QTL were detected using WinQTL Cartographer (http://statgen.ncsu.edu/qtlcart/WQTLCart.htm).

Measures of plant height, basal tiller number and node number on the main stem were correlated; therefore multiple-trait composite interval mapping was performed to estimate QTL effects. Likelihood thresholds were determined by simulation (500 random permutations of genotypes among individuals) and included a Bonferroni correction for multiple testing.

### Polymorphism survey and selection tests

The phenotype of sequenced accessions was checked under controlled greenhouse conditions prior to DNA extraction. PCR products were cloned and sequenced using the TOPO-TA (Invitrogen) and ABI-Prism v3.0 kits, assembled using the Staden package and aligned using ClustalW. Two independent products per individual were sequenced and a site was tagged polymorphic if it was found consistently so in both products. Sequences were deposited in Genbank under accession numbers EF694113-EF694165 (*PgTb1*), GQ472665-GQ472771 and JN125251-JN125254 (STS loci). Sorghum sequences used for the HKA (XM 002450113.1 and AF466204) test were retrieved from Genebank by using a BLASTn search. Trees were constructed with MEGA [53], polymorphism and molecular evolution analyses were performed using DnaSP [54] and the MS program [55] to simulate demographic models by coalescence methods. Statistics were computed from the outputs of the MS program using FABSIM [56].

#### Rationale for the coalescence simulations to test for selection

Patterns of polymorphism were tested for selection using a modified Tajima's D and Fu &Li's F* tests [36], [37]. In their classic version, these tests assume a large and constant population size. While this could be a reasonable hypothesis for wild *P.glaucum* (Figure S3A), it is unrealistic for domesticated landraces. Accordingly, we adapted these tests to include demography: following the rationale of Hudson's haplotype test of selection, we used coalescence simulations to infer the distribution of D and F* expected from a bottleneck and subsequent expansion, without selection (Figure S3B). The probability of the D and F* values observed in the domesticated sample was deduced from this null distribution.

The archaeological record of pearl millet domestication [38], [39] was accounted for in the parameters of these demographic models, which were varied one at a time in a combination of 54 scenarios listed in Table S3 (see below for details). They explored a wide range of possible demographic histories.

#### Implementation of demographic models with the MS program

The parameters of coalescence models are detailed in Figure S3B. They explored a range of possible bottleneck lengths (100 or 500 years, as a proxy to the duration of the domestication process), bottleneck intensity (a 5%, 0.5% or 0.05% ratio of population size, as a proxy to selection intensity) and expansion strength (100-, 10 or 1-fold expansion respective to initial population size). Population size variation was assumed instantaneous for the coalescence simulation but we observed no difference by simulating an exponential growth (data not shown). We also tested a bottleneck of 1000 years with no significant differences in the conclusions for selection tests (data not shown). Models also took into account different possible mutation rates for the *PgTb1* locus (equivalent to the *Adh1* locus, 10 times less or 10 times more). Specifically, the MS program command line (Figure S3B) required the sample size and number of segregating sites S (fixed for each locus/ *PgTb1* region and reported in Table 1), the time for the end of the bottleneck (fixed to t1 = 4,000 years according to archaeological data for the completion of pearl millet domestication), the time for the beginning of the bottleneck (t2 = 4100 or 4500 years), the ratio of population size during the bottleneck (N_1_/N_2_, 3 possible values), the ratio of population size during the expansion (N_0_/N_2_, 3 possible values). The mutation rate µ (3 possible values) and Watterson's estimator θ (Table 1) were used to convert time points t1 and t2 from years to units of 4N_0_, as described in the documentation of the MS program [55]. Each variable parameter was then changed one at a time, resulting in 54 combinations (scenarios) listed in Table S3. Ten thousand simulations of each scenario were run, assuming absence of recombination. The rejection tests for selective neutrality of nucleotide polymorphisms in *Tb1* and the three STS loci in the domesticated sample were done by using each of the 54 distributions of D and F* generated for the 54 scenarios.

#### Testing the fit of demographic scenarios

Our objective was not to infer the history of pearl millet domestication because we considered that three potentially neutral loci would be not enough to reach this goal. Rather, our goal was to compare our *PgTb1* data to the neutral expectations under a wide range of possible demographic scenarios. Nevertheless, we estimated the approximate likelihood of these 54 scenarios from the STS data (see below and Figure S3C for details). To evaluate the likelihood of these scenarios, we followed the same method as implemented by Tenaillon et al. in maize [57]. For each STS locus and each scenario, we computed the frequency of simulations (among the 10,000 repeats) for which summary statistics were included into the range +/− 10%, 20% or 30% of the observed values of these statistics. Both π_Dom_ and the ratio π_Dom_/π_Wild_ were used as summary statistics. A multi-locus approximate likelihood of each scenario was estimated by multiplying the individual frequencies obtained for the 3 STS loci. Likelihood values for all 54 scenarios are plotted in Figure S3C. Results obtained with the two summary statistics were very similar for all three ranges and all scenarios. Those obtained for the π_Dom_/π_Wild_ ratio are given in Figure S3C. From these results, only scenario 39 (long and intense bottleneck, large expansion, high mutation rate) was subsequently discarded as highly unlikely relative to the others. Other scenarios were otherwise roughly equally plausible and, therefore, were all taken into account to test for neutrality in the domesticated sample. This was in agreement with previous observations from coalescence modeling of demography and domestication in maize [58] for which variation in individual parameters had little influence on the likelihood of models. Under each scenario, the *p*-values of D and F* were calculated: significant *p*-values consistent across all possible demographic models (*ie* regardless of the true demographic history of pearl millet domestication) were considered to provide a strong support in favor of selection.

## Supporting Information

Figure S1 **RT-PCR evidence of PgTb1 expression.** Electrophoresis of the RT-PCR product issued from amplification on pearl millet cDNA (and control DNA and water), using the primers represented by horizontal arrows on the schematic of the *PgTb1* gene structure.
(TIF)

Figure S2 **Polymorphism survey.** A. Schematic diagram of the *PgTb1* region sequenced for the polymorphism survey. B. Map of accessions sequenced for the *PgTb1* polymorphism survey
(TIF)

Figure S3 **Demographic models simulated by coalescence methods for tests of selection.** A. Fisher-Wright constant size population model for wild *P.glaucum*. The command line for the coalescence simulation by the MS coalescence program, and associated parameters are indicated to the right. B. Bottleneck followed by an instantaneous population expansion for domesticated pearl millet. The different demographic parameters tested are indicated in the table to the right, as well as the specific parameters and command line for the coalescence simulation by the MS program. C. Multi-locus approximate log-likelihood of each demographic scenario. Approximate likelihood was estimated based on the proportion of the 10,000 simulations for which all of the π dom/π wild ratio was within 10%, 20% or 30% of their observed values in the STS loci (see Methods for details). Scenarios are numbered 1-54 as listed in Table S3.
(TIF)

Figure S4 **Conservation of the expression pattern of Teosinte-branched1 orthologues in pearl millet (** ***P.glaucum*** **) and sorghum (** ***Sorghum bicolor*** **).** In sorghum (right) like in pearl millet (left), vegetative branching is reduced because axillary meristems remain dormant (arrows). In-situ hybridization in serial transverse sections of 10 day-old seedlings shows that Tb1 is expressed in axillary meristems in both species (a–c: pearl millet;d–f: sorghum).
(TIF)

Table S1 **List of primers used in this study.**
(PDF)

Table S2 **List of accessions sequenced for the PgTb1 polymorphism study.**
(PDF)

Table S3 ***p-*** **values of neutrality tests in the domesticated sample for each simulated demographic scenario.** Those scenarios rejected according to their likelihood scores are in italics.
(PDF)
