# Supplementary figures and images for: Cereal Domestication and Evolution of Branching: Evidence for Soft Selection in the Tb1 Orthologue of Pearl Millet (Pennisetum glaucum [L.] R. Br.)
Source: PLoS One. 2011 Jul 22;6(7):e22404. doi: 10.1371/journal.pone.0022404 (PMC3142148; doi:10.1371/journal.pone.0022404)

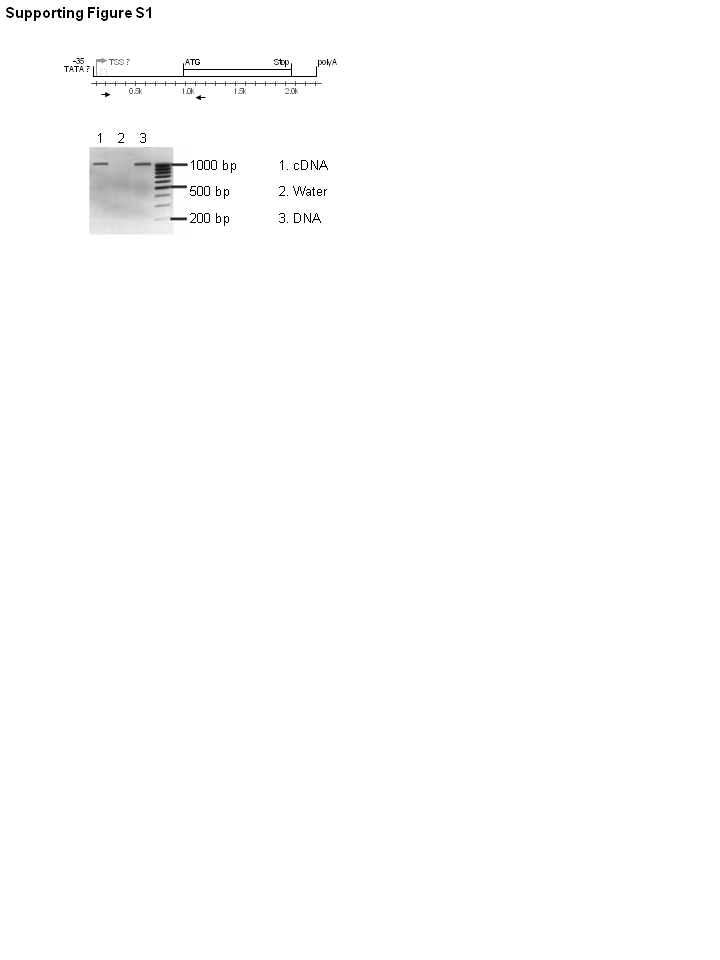

Supplement: Figure S1 — RT-PCR evidence of PgTb1 expression. Electrophoresis of the RT-PCR product issued from amplification on pearl millet cDNA (and control DNA and water), using the primers represented by horizontal arrows on the schematic of the PgTb1 gene structure. (TIF) [file pone.0022404.s001.tif]

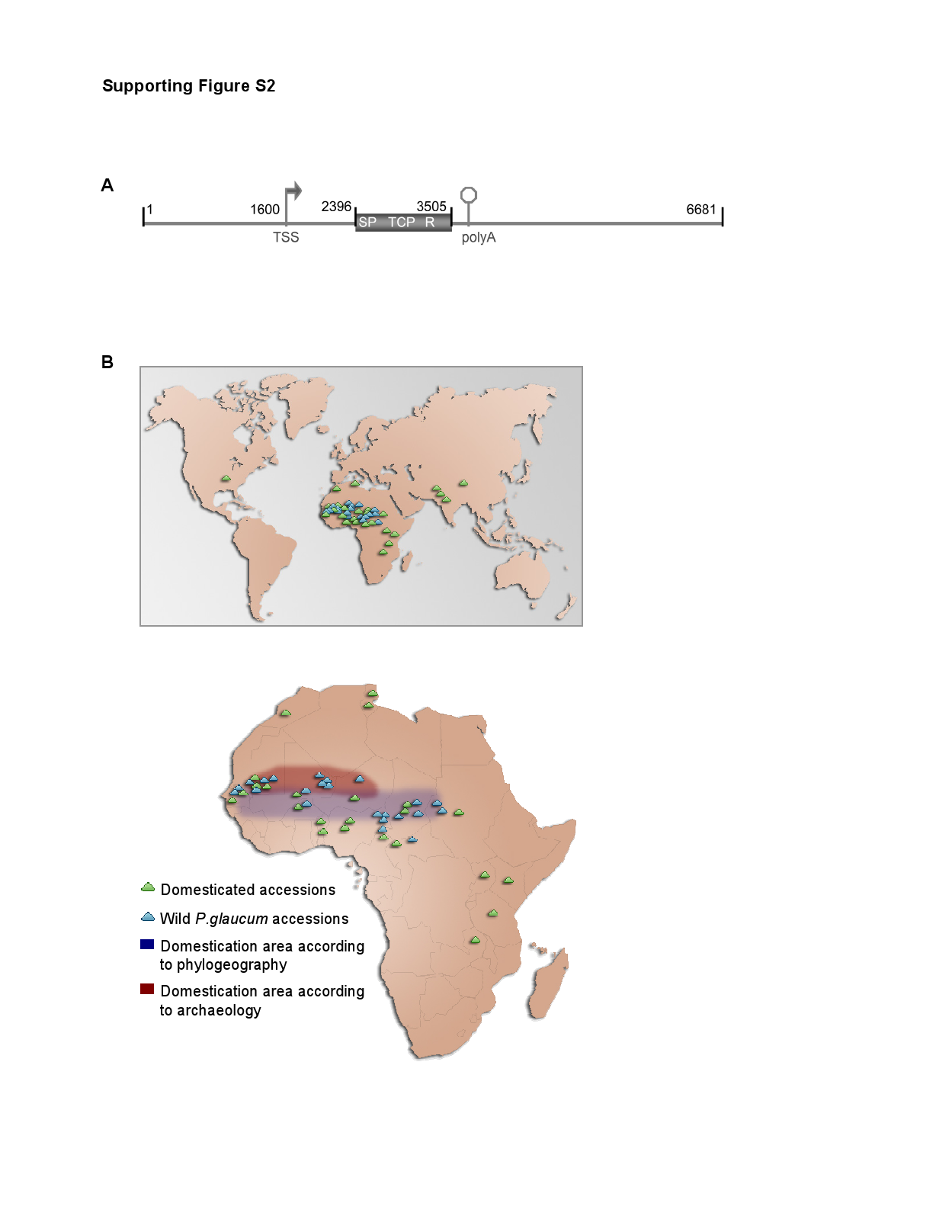

Supplement: Figure S2 — Polymorphism survey. A. Schematic diagram of the PgTb1 region sequenced for the polymorphism survey. B. Map of accessions sequenced for the PgTb1 polymorphism survey (TIF) [file pone.0022404.s002.tif]

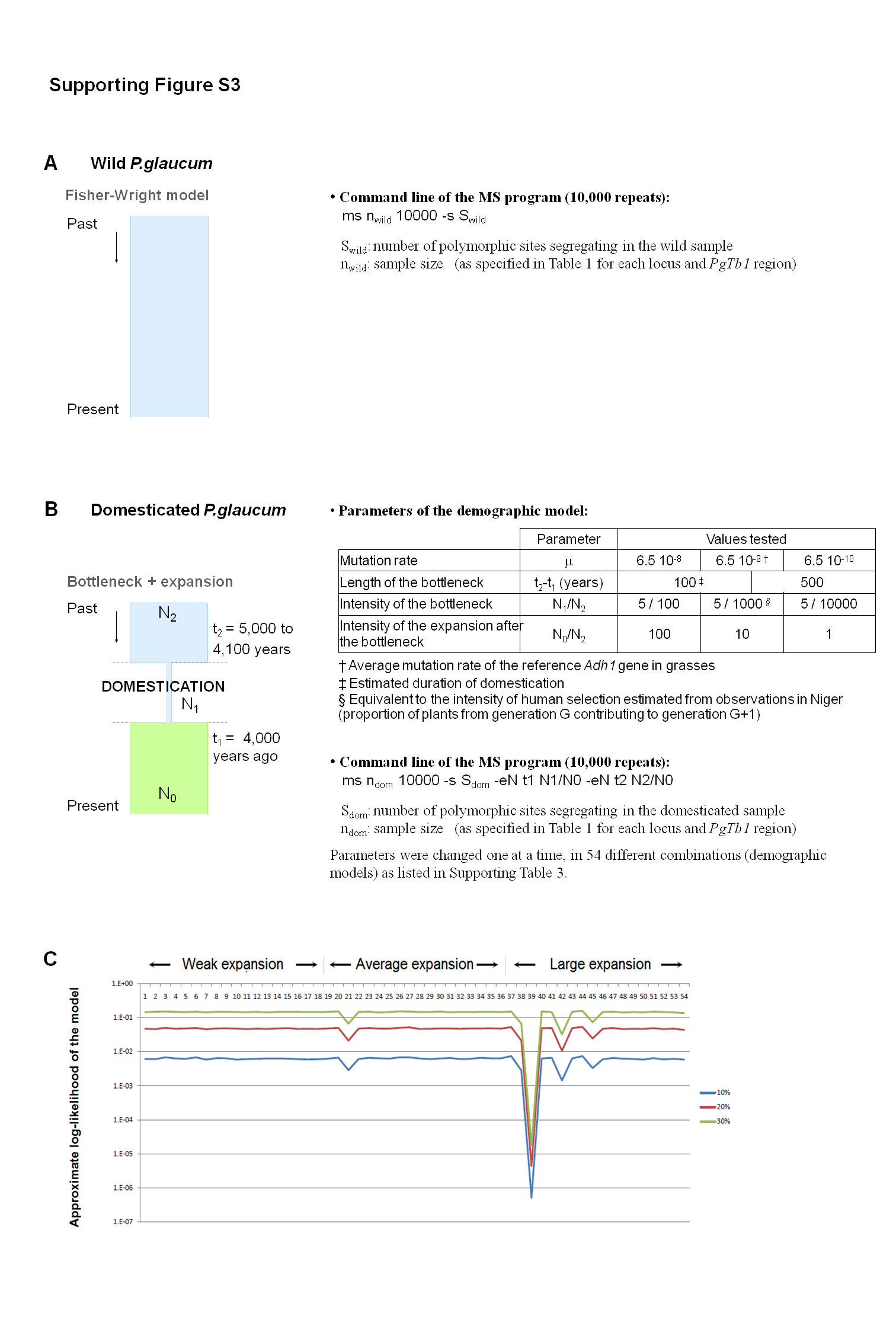

Supplement: Figure S3 — Demographic models simulated by coalescence methods for tests of selection. A. Fisher-Wright constant size population model for wild P.glaucum. The command line for the coalescence simulation by the MS coalescence program, and associated parameters are indicated to the right. B. Bottleneck followed by an instantaneous population expansion for domesticated pearl millet. The different demographic parameters tested are indicated in the table to the right, as well as the specific parameters and command line for the coalescence simulation by the MS program. C. Multi-locus approximate log-likelihood of each demographic scenario. Approximate likelihood was estimated based on the proportion of the 10,000 simulations for which all of the π dom/π wild ratio was within 10%, 20% or 30% of their observed values in the STS loci (see Methods for details). Scenarios are numbered 1-54 as listed in Table S3. (TIF) [file pone.0022404.s003.tif]

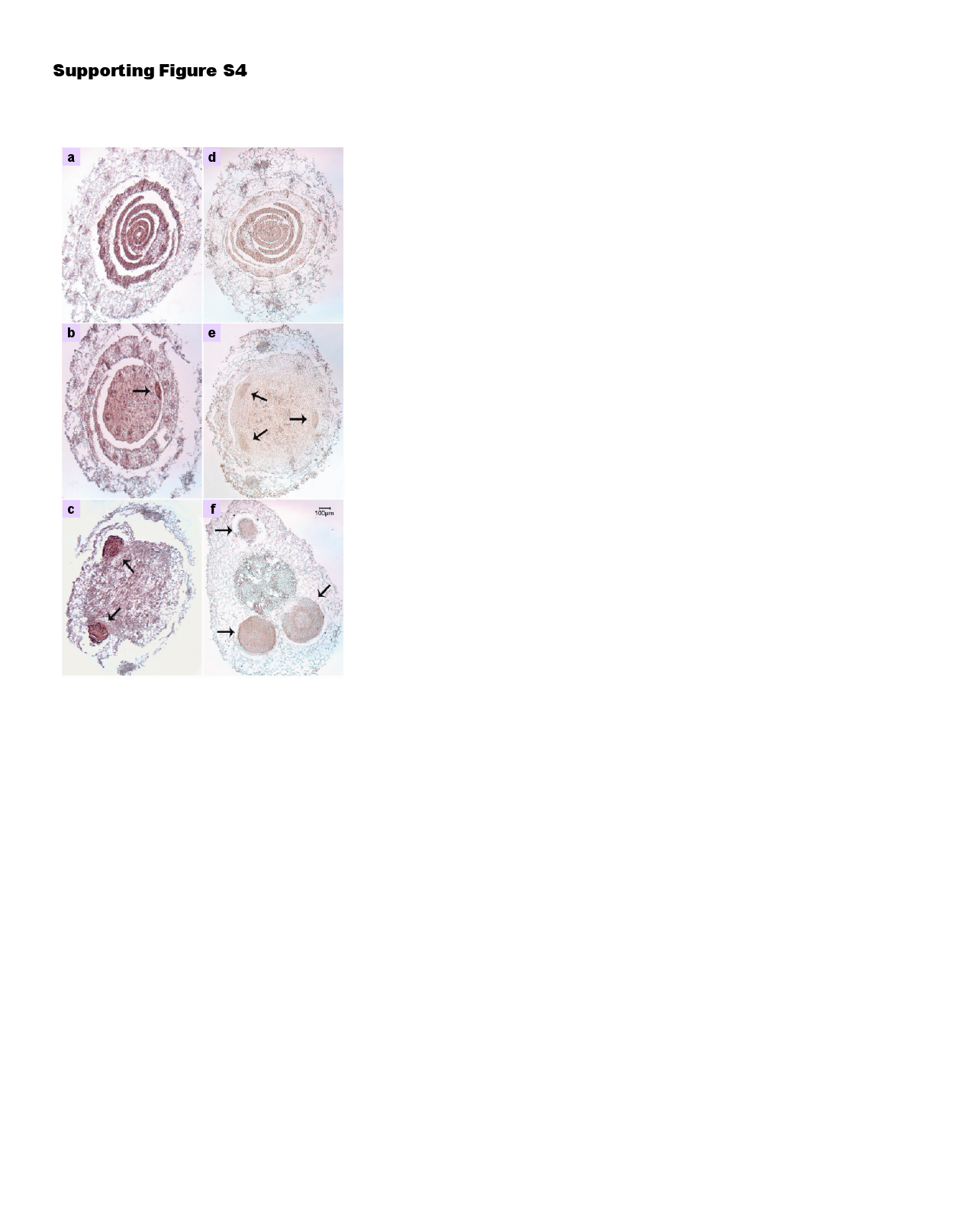

Supplement: Figure S4 — Conservation of the expression pattern of Teosinte-branched1 orthologues in pearl millet ( P.glaucum ) and sorghum ( Sorghum bicolor ). In sorghum (right) like in pearl millet (left), vegetative branching is reduced because axillary meristems remain dormant (arrows). In-situ hybridization in serial transverse sections of 10 day-old seedlings shows that Tb1 is expressed in axillary meristems in both species (a–c: pearl millet;d–f: sorghum). (TIF) [file pone.0022404.s004.tif]
