# Supplementary material for: Cereal Domestication and Evolution of Branching: Evidence for Soft Selection in the Tb1 Orthologue of Pearl Millet (Pennisetum glaucum [L.] R. Br.)
Source: PLoS One. 2011 Jul 22;6(7):e22404. doi: 10.1371/journal.pone.0022404 (PMC3142148; doi:10.1371/journal.pone.0022404)
Supplement: Table S1 — List of primers used in this study. (PDF) [file pone.0022404.s005.pdf]

Supporting Table 1. List of primers used in the study.

PgTb1 primers	
Name	Sequence
**Cloning of *PgTb1***	
U1	TGCACTGGATCTATCACTACTCA
L2	AGTTCATCGTCACACAGCCAATTAC
**Southern blotting and library hybridization; *PgTb1* mapping**	
U1	TGCACTGGATCTATCACTACTCA
L1	GAGTCGATCACGGCTTGTG
**RT-PCR analysis of the structure of Tb1**	
F1	ATGAGCCCATGCCTCCTC
F2	CATACAGGTCCCATATGCCTAA
R1	GCTGTTGGTAAAGCGGTAAGTC
R2	GGCATGGGCTCATGTTCATAC
***In-situ* hybridization probe**	
In-situF	CTCCAGCAGCCTCTCCGT
In-situR	ATGTTGGATGATGGGTGGT
**Polymorphism in worldwide collection (numbers refer to position in the reference Tift23DB**	
PCR amplification	
53F	AACCTGTCCAGCTCCTCACAAA
1818R	ACTGAACACCGTGGGAGTGT
1703F	GCCTAAAGTTGGGAAGACCAG
4266R	CATGGTAGAGAAATTGACCTTCAG
3380F	GGTGCTCATCAACCCTCAAC
6693R	ACAATTGCCTTGCATTGGTC
Sequencing	
453F	AATGAAAGGATAAGACCAGGAAA
860F	CCCATTTTTGGAACAACACC
908R	TACGTACGCAAATTCTGTTG
1260F	ATAGGTCTGAATGCCACGTTTC
1756R	ATGGTAGGGGACCGATGTG
1765F	ACACTACGACAAAAAGCACTGG
2171F	TGCACTTGATGTCTTGATGC
2523F	CAATCCACCACCAGCTTCTT
2604R	GCTGGTAGCTGAGGTGGAAG
2987F	CTCCAGCAGCCTCTCCGT
3410R	TTGCATCTTCCCTCCATTGT
4174F	ATGATTCAATTGTTCGATGAAAG
4266R	CATGGTAGAGAAATTGACCTTCAG
4667F	ATCATGCAGTTTCAGTTTCTGT
4970F	GATGTTGGCATATCTTAATTTTGTC
5077R	AAATGCACAAAGCACCTGAA
5447F	TGGATGACCGGATGTGAAC
5788F	ACTTGAGGATGCAAACTGACAC
5839R	GATGTATAGTGAACCACCCTCTG
6257F	CTTGCTTGGTTAATCCCTTTCA

STS loci primers	
Name	Sequence
STS713F	CTAGTCGAAGCATTGTGAATC
STS713R	TCTGGATGCACCATAACC
STS738F	CATCAGCATGTGAGCATG
STS738R	TCCACAGTGAGCAACCTCAG
STS476F	GACCGAGCCTCCTTTCCTG
STS476R	CGTTCTAGCATTCGGGATTATGG

EF1-alpha primers	
Name	Sequence
EF1A-F1	TTACAGATGGATGCTACTACACCCAAG
EF1A-R1	ATCTGGTCAAGAGCCTCAAGCA
EF1A-R2	TTGTACCAGTCAAGGTTGGTGGA
EF1A-R3	ATGGGGACAAAGGCAATCTTGTA
