# Supplementary material for: Cereal Domestication and Evolution of Branching: Evidence for Soft Selection in the Tb1 Orthologue of Pearl Millet (Pennisetum glaucum [L.] R. Br.)
Source: PLoS One. 2011 Jul 22;6(7):e22404. doi: 10.1371/journal.pone.0022404 (PMC3142148; doi:10.1371/journal.pone.0022404)
Supplement: Table S2 — List of accessions sequenced for the PgTb1 polymorphism study. (PDF) [file pone.0022404.s006.pdf]

Supporting table S2. List of accessions sequenced for the PgTb1 polymorphism study.

Name	Type of the population collected	Phenotype of the individual	Geographic origin	sequenced for STS713	sequenced for STS738	sequenced for STS476
Tb1	Domesticated	Domesticated	USA, Tift23DB cultivar			+
Tb5	Domesticated	Domesticated	Benin			+
Tb6	Domesticated	Domesticated	Benin			
Tb11	Domesticated	Domesticated	Burkina-faso			+
Tb13	Wild	Wild	Burkina-faso	+	+	
Tb16	Domesticated	Domesticated	Cameroon	+	+	
Tb18	Wild	Wild	Cameroon	+		+
Tb19	Wild	Wild	Cameroon			
Tb70	Wild	Weedy	Centrafrican Republic	+	+	+
Tb71	Domesticated	Domesticated	Centrafrican Republic			+
Tb90	Domesticated	Domesticated	Chad		+	+
Tb92	Domesticated	Domesticated	Chad	+	+	
Tb94	Wild	Wild	Chad	+		+
Tb96	Wild	Wild	Chad			
Tb89	Domesticated	Domesticated	Chad	+		+
Tb20	Domesticated	Domesticated	China	+		+
Tb68	Domesticated	Domesticated	India (rajasthan)			+
Tb27	Domesticated	Domesticated	India, cultivar J104	+		+
Tb28	Domesticated	Domesticated	Kenya	+		+
Tb31	Domesticated	Domesticated	Mali		+	+
Tb37	Wild	Wild	Mali			+
Tb38	Wild	Wild	Mali	+		+
P8470	Wild	Wild	Mali	+	+	+
P8473	Wild	Wild	Mali	+	+	+
P8475	Wild	Wild	Mali	+	+	+
Tb42	Domesticated	Domesticated	Mauritania	+	+	+
Tb43	Domesticated	Domesticated	Mauritania	+	+	+
Tb45	Domesticated	Domesticated	Mauritania	+	+	+
Tb46	Wild	Wild	Mauritania	+		
Tb47	Wild	Wild	Mauritania		+	
P8493	Wild	Wild	Mauritania	+	+	+
P8497	Wild	Wild	Mauritania	+	+	+
Tb40	Domesticated	Domesticated	Morocco	+	+	+
Tb49	Domesticated	Domesticated	Niger			
Tb52	Wild	Wild	Niger	+	+	+
Tb56	Domesticated	Domesticated	Nigeria	+	+	+
Tb57	Domesticated	Domesticated	Nigeria	+	+	+
Tb59	Wild	Wild	Nigeria	+	+	+
Tb61	Wild	Wild	Nigeria	+		
Tb67	Domesticated	Domesticated	Pakistan	+		+
Tb75	Domesticated	Domesticated	Senegal	+		+
Tb78	Wild	Wild	Senegal	+		+
Tb79	Wild	Wild	Senegal	+	+	
Tb74	Domesticated	Domesticated	Senegal	+	+	+
Tb80	Domesticated	Domesticated	Sudan			+
Tb83	Wild	Weedy	Sudan	+		+
Tb84	Wild	Weedy	Sudan			
Tb88	Domesticated	Domesticated	Tanzania	+		+
Tb99	Domesticated	Domesticated	Tunisia	+	+	+
Tb100	Domesticated	Domesticated	Tunisia			+
Tb62	Domesticated	Domesticated	Uganda	+	+	
Tb103	Domesticated	Domesticated	Zambia		+	+
			Additional accessions (for STS)	95 W chad<br>P8491 W Mauritania<br>Alzu-W-Niger	P8491 W mauritania<br>P8475S- W- Mali<br>TB59BS-W-Nigeria<br>TB16S-D-Cameroon<br>TB31BC-D-Mali<br>TB42BC-D-Mauritania	7 D benin<br>25 D India<br>30 D mali<br>44 D Mauritania
