# Supplementary material for: Cereal Domestication and Evolution of Branching: Evidence for Soft Selection in the Tb1 Orthologue of Pearl Millet (Pennisetum glaucum [L.] R. Br.)
Source: PLoS One. 2011 Jul 22;6(7):e22404. doi: 10.1371/journal.pone.0022404 (PMC3142148; doi:10.1371/journal.pone.0022404)
Supplement: Table S3 — p- values of neutrality tests in the domesticated sample for each simulated demographic scenario. Those scenarios rejected according to their likelihood scores are in italics. (PDF) [file pone.0022404.s007.pdf]

Supporting Table S3: *p*-values of neutrality tests in the domesticated sample for each simulated demographic scenario. Those scenarios rejected according to their likelihood scores are in italics.

					p-value for Tajima's D (in %)						p-value for Fu & Li's F* (in %)						p-value for Fu's Fs (in %)					
					PgTb1 upstream	*PgTb1* mRNA	*PgTb1* downstream	STS 713	STS 738	STS 476	*PgTb1* upstream	*PgTb1* mRNA	*PgTb1* downstream	STS 713	STS 738	STS 476	*PgTb1* upstream	*PgTb1* mRNA	*PgTb1* downstream	STS 713	STS 738	STS 476
**Weak expansion (N0/N2 =1)**	Intense bottleneck (N1/N2 = 5e-4)	Long bottleneck (500y)	u weak	Model 1	0.11	0.15	< 0.01	5.3	51.8	51.3	0.39	0.06	< 0.01	5.1	29.3	44.8	0.24	0.04	< 0.01	3.08	34.00	7.40
			u average	Model 2	0.08	0.12	0.03	5.2	51.7	49.1	0.35	0.07	0.01	5.2	28.2	41.1	0.20	0.02	< 0.01	2.84	33.02	6.14
			u strong	Model 3	0.14	0.07	< 0.01	4.4	48.3	33.3	0.34	0.06	0.01	4.3	24.7	22.3	0.02	< 0.01	< 0.01	0.91	23.45	0.87
		Short bottleneck (100y)	u weak	Model 4	0.06	0.11	0.01	5.6	51.4	51.6	0.29	0.07	< 0.01	5.4	28.3	44.7	0.22	0.05	< 0.01	3.17	34.07	7.54
			u average	Model 5	0.12	0.12	< 0.01	5.5	51.2	50.8	0.35	0.11	0.01	5.4	28.7	43.8	0.11	0.01	< 0.01	3.00	33.15	7.12
			u strong	Model 6	0.09	0.05	< 0.01	5.2	50.8	47.4	0.25	0.06	< 0.01	5.3	27.9	38.4	0.16	0.05	< 0.01	2.42	31.42	5.15
	Average bottleneck (N1/N2 = 5e-3)	Long bottleneck (500y)	u weak	Model 7	0.1	0.06	< 0.01	5.3	52	51.3	0.33	0.05	< 0.01	5.3	28.4	45	0.13	0.02	< 0.01	3.06	34.25	7.75
			u average	Model 8	0.13	0.1	< 0.01	5.4	51.5	50.8	0.41	0.06	< 0.01	5.4	28.8	44.4	0.20	0.05	< 0.01	2.73	33.64	7.47
			u strong	Model 9	0.09	0.07	0.01	5.6	51.1	49.7	0.3	0.08	< 0.01	5.4	28.4	41.2	0.14	0.03	< 0.01	2.59	32.68	6.13
		Short bottleneck (100y)	u weak	Model 10	0.11	0.11	< 0.01	5.4	50.9	51.3	0.28	0.08	< 0.01	5.3	28.2	44.6	0.18	0.04	< 0.01	3.24	32.85	7.20
			u average	Model 11	0.18	0.09	0.02	5.7	51.8	52	0.38	0.07	< 0.01	5.4	28.6	45	0.17	0.03	< 0.01	3.52	33.60	7.72
			u strong	Model 12	0.04	0.09	0.01	5.4	51.1	51.4	0.23	0.05	< 0.01	4.9	28.7	43.6	0.18	0.05	< 0.01	3.06	33.82	6.89
	Weak bottleneck (N1/N2 = 5e-2)	Long bottleneck (500y)	u weak	Model 13	0.13	0.05	0.02	5.7	51.5	50.9	0.31	0.03	< 0.01	5.6	28.4	45	0.19	0.11	< 0.01	3.02	33.02	7.52
			u average	Model 14	0.06	0.08	< 0.01	5.4	50.7	51.6	0.2	0.08	< 0.01	5.3	28	44.6	0.27	0.04	< 0.01	2.86	33.59	7.08
			u strong	Model 15	0.09	0.09	0.02	5.3	51.3	51.1	0.32	0.08	< 0.01	5.1	28.7	44.2	0.21	0.04	< 0.01	2.96	32.79	7.33
		Short bottleneck (100y)	u weak	Model 16	0.11	0.11	< 0.01	5.3	52	50.7	0.25	0.07	< 0.01	5.2	28.6	44.1	0.24	0.03	< 0.01	3.01	33.52	7.55
			u average	Model 17	0.09	0.08	0.01	5.4	51.3	51.1	0.37	0.08	< 0.01	5.3	28	44.7	0.09	0.01	< 0.01	2.94	33.79	7.45
			u strong	Model 18	0.12	0.09	0.01	5.4	52.1	51.1	0.31	0.06	< 0.01	5.3	29.4	44.7	0.21	0.06	< 0.01	2.91	33.94	7.50
**Average expansion (N0/N2 =10)**	Intense bottleneck (N1/N2 = 5e-4)	Long bottleneck (500y)	u weak	Model 19	0.13	0.07	< 0.01	5.1	51.5	48.9	0.34	0.06	< 0.01	5.1	28.3	40.9	0.27	0.02	< 0.01	2.68	32.58	5.91
			u average	Model 20	0.11	0.08	< 0.01	4.2	48.1	32.7	0.28	0.08	< 0.01	4	25.6	21.7	0.02	0.01	< 0.01	0.91	23.99	0.86
			u strong	*Model 21*	*0.09*	*0.11*	*< 0.01*	*3.9*	*29*	*27.8*	*0.22*	*0.1*	*< 0.01*	*4.6*	*14.2*	*18.5*	*< 0.01*	*< 0.01*	*< 0.01*	*< 0.01*	*0.23*	*5.80*
		Short bottleneck (100y)	u weak	Model 22	0.11	0.08	0.01	5.3	51.4	50.7	0.39	0.09	< 0.01	5.1	28.1	44.6	0.16	0.03	< 0.01	2.78	33.40	7.15
			u average	Model 23	0.05	0.11	< 0.01	5	51	46.3	0.3	0.09	< 0.01	5	28	37.3	0.10	0.03	< 0.01	2.38	31.60	5.08
			u strong	Model 24	0.08	0.07	0.03	4.4	44.7	25.7	0.24	0.04	0.02	4.5	22.4	16.9	0.00	< 0.01	< 0.01	0.31	15.66	0.27
	Average bottleneck (N1/N2 = 5e-3)	Long bottleneck (500y)	u weak	Model 25	0.09	0.1	< 0.01	5.3	51.1	50.6	0.33	0.03	0.01	5.1	28.3	44.3	0.26	0.01	< 0.01	2.86	33.48	7.01
			u average	Model 26	0.11	0.07	0.01	5.7	51.2	48.5	0.28	0.04	< 0.01	5.8	27.9	40.5	0.15	0.03	< 0.01	2.62	32.48	5.78
			u strong	Model 27	0.05	0.05	0.03	4.5	48.3	34	0.19	0.03	0.01	4.7	25.3	24.1	0.03	< 0.01	< 0.01	0.94	23.92	1.20
		Short bottleneck (100y)	u weak	Model 28	0.07	0.09	0.02	5.1	52.4	51.3	0.26	0.09	0.01	4.9	29.1	45	0.25	0.05	< 0.01	3.04	33.52	7.24
			u average	Model 29	0.12	0.12	0.01	5.7	50.9	50.9	0.28	0.08	0.01	5.5	28.5	43.9	0.19	0.04	< 0.01	3.13	32.51	7.16
			u strong	Model 30	0.03	0.13	0.01	5.3	50.3	47.5	0.22	0.08	< 0.01	5.3	27.5	38.8	0.18	0.02	< 0.01	2.68	31.62	5.29
	Weak bottleneck (N1/N2 = 5e-2)	Long bottleneck (500y)	u weak	Model 31	0.09	0.07	< 0.01	5.5	51.2	52.2	0.27	0.04	< 0.01	5.5	28.6	45.9	0.25	0.08	< 0.01	3.04	33.75	7.40
			u average	Model 32	0.12	0.1	0.01	5.4	52.1	52.4	0.27	0.09	< 0.01	5.4	29.2	45.1	0.26	0.01	< 0.01	2.94	34.27	7.47
			u strong	Model 33	0.1	0.04	< 0.01	5.7	51.2	50.2	0.3	0.03	< 0.01	5.5	28.4	43.3	0.23	0.02	< 0.01	2.68	32.63	6.57
		Short bottleneck (100y)	u weak	Model 34	0.11	0.12	< 0.01	5.5	51.1	51.8	0.3	0.07	< 0.01	5.5	28.4	45.4	0.22	0.04	< 0.01	3.04	33.17	7.20
			u average	Model 35	0.12	0.12	0.02	5.7	51.7	51	0.34	0.04	0.02	5.3	28.8	44.9	0.21	0.05	< 0.01	3.16	33.46	7.00
			u strong	Model 36	0.1	0.12	< 0.01	5.4	51.9	51.6	0.41	0.07	< 0.01	5	29	46.5	0.17	0.06	< 0.01	3.07	34.65	7.86
**Large expansion (N0/N2 =100)**	Intense bottleneck (N1/N2 = 5e-4)	Long bottleneck (500y)	u weak	Model 37	0.11	0.06	< 0.01	4.6	47.8	32.9	0.26	0.07	< 0.01	4.5	25.1	22.3	0.04	0.00	< 0.01	1.09	23.80	0.86
			u average	*Model 38*	*0.13*	*0.14*	*< 0.01*	*4*	*29*	*27.1*	*0.31*	*0.17*	*< 0.01*	*4.5*	*13.7*	*17.9*	*0.00*	*0.00*	*< 0.01*	*0.01*	*0.19*	*5.48*
			u strong	*Model 39*	*14.72*	*46.7*	*2.73*	*74.3*	*50.6*	*99.94*	*15.47*	*47.1*	*2.9*	*74.1*	*40.2*	*99.98*	*11.72*	*43.37*	*0.90*	*70.09*	*32.74*	*98.71*
		Short bottleneck (100y)	u weak	Model 40	0.04	0.12	0.01	5.1	50	46.6	0.2	0.05	< 0.01	4.9	27.3	37.9	0.14	0.02	< 0.01	2.49	30.92	4.64
			u average	Model 41	0.06	0.04	< 0.01	3.8	45	26.5	0.21	0.02	< 0.01	4	22.8	17.9	< 0.01	< 0.01	< 0.01	0.28	16.27	0.21
			u strong	*Model 42*	*0.13*	*0.57*	*0.04*	*7.1*	*24.6*	*71.5*	*0.65*	*0.61*	*0.02*	*7.9*	*13.1*	*75.9*	*0.01*	*< 0.01*	*< 0.01*	*0.88*	*0.18*	*49.14*
	Average bottleneck (N1/N2 = 5e-3)	Long bottleneck (500y)	u weak	Model 43	0.11	0.09	< 0.01	4.7	51.6	49.2	0.39	0.04	< 0.01	4.7	28.5	41.8	0.16	0.06	< 0.01	2.61	33.32	6.13
			u average	Model 44	0.08	0.06	0.01	4.8	48.3	33.9	0.32	< 0.01	0.01	4.4	24.6	23.9	0.03	0.00	< 0.01	1.20	23.50	1.33
			u strong	*Model 45*	*0.1*	*0.18*	*< 0.01*	*4*	*31.5*	*47.3*	*0.37*	*0.18*	*< 0.01*	*4.8*	*15.2*	*52.5*	*< 0.01*	*< 0.01*	*< 0.01*	*0.00*	*1.04*	*14.08*
		Short bottleneck (100y)	u weak	Model 46	0.09	0.08	0.02	5.2	52	51.7	0.29	0.09	0.01	4.9	29.5	45	0.13	0.06	< 0.01	3.28	34.06	7.09
			u average	Model 47	0.12	0.06	< 0.01	5.2	51.2	48	0.38	0.04	< 0.01	4.9	28.8	39.4	0.20	0.01	< 0.01	3.02	32.28	5.24
			u strong	Model 48	0.14	0.05	< 0.01	4.3	47.1	40.1	0.35	0.05	< 0.01	4.5	24.2	37.8	0.02	< 0.01	< 0.01	0.88	20.29	3.17
	Weak bottleneck (N1/N2 = 5e-2)	Long bottleneck (500y)	u weak	Model 49	0.1	0.09	< 0.01	5.1	52.1	51.9	0.36	0.05	< 0.01	5.1	29.7	45.5	0.22	0.02	< 0.01	2.84	34.11	7.57
			u average	Model 50	0.11	0.09	0.01	5.2	51.3	50.9	0.27	0.05	< 0.01	5.1	28.3	43.4	0.16	0.03	< 0.01	2.79	32.78	6.64
			u strong	Model 51	0.11	0.08	< 0.01	5.3	49.5	46.6	0.25	0.05	0.01	5.3	26.3	42.7	0.13	0.01	< 0.01	1.82	29.23	5.78
		Short bottleneck (100y)	u weak	Model 52	0.12	0.08	0.01	5.4	51.2	51.6	0.33	0.07	< 0.01	5.3	28.7	45.5	0.13	0.05	< 0.01	3.16	34.00	7.23
			u average	Model 53	0.12	0.13	0.01	5.4	52.2	51.9	0.37	0.09	< 0.01	5.2	29.3	45.6	0.23	0.04	< 0.01	3.37	35.27	8.38
			u strong	Model 54	0.16	0.06	< 0.01	5.6	52.9	57.3	0.34	0.05	< 0.01	5.4	30.3	54.4	0.32	0.06	< 0.01	3.95	36.79	11.21
